# Supplementary material for: Genome-Wide Identification and Transcriptome-Based Expression Profile of Cuticular Protein Genes in Antheraea pernyi
Source: Int J Mol Sci. 2023 Apr 10;24(8):6991. doi: 10.3390/ijms24086991 (PMC10138643; doi:10.3390/ijms24086991)
Supplement: Supplementary file 1 [file ijms-24-06991-s001.zip › Figure S3.pdf]

CPAP1:

```
ApCPAP1-A  : ----------------------------------------------QAVIRKPLREHEKPQDLRNVPGTPGVDFPIYHTVP---------------------ETGFSCAH------------VPVHPGMYA :  91
ApCPAP1-B  : LTD-KQSTSTIAPDHDKTEDIYIPDEPYLAAVKNPSQHIDDFQP--GEGTIQRFPLPITSDEEGGTPGRPGIDYPTLTTIP---------------------QTVFICK-------------TQRYKGFFA : 107
ApCPAP1-C  : ----------------------------------------------------------------------------------------------------------------------------------M :   1
ApCPAP1-D  : ----------------------------------------------------MSQQPIDYYHDLHLPHDPPL-HPVFEHAP---------------------KTDFSCA--------------GRHRGYYA :  43
ApCPAP1-F  : ----------------------------------------------FGLLAVFMYLVDRSFGQALDDFVPGEDYPVFTEVP--------------------KGLSFTCD--------------DKIPGYYA :  64
ApCPAP1-I  : ----------------------------------------------PEYRAGGVRASTGQSLRDALRDTMSPVWLLLILLP-----------------VLGGAERFNCK-------------TRILGAYYP :  72
ApCPAP1-J  : ----------------------------------------------KRLIPADSQPDLDVVEHQGVIGKAGVDFPAFPNIP---------------------NTGFICK--------------NAPTGYYA : 116
ApCPAP1-K  : -----------STSTTSTTSTTEPPVLFEPEPEILLTAVPQKPQ--RLPTSGKVMNAPKEYYPVGYEKNFDDHFQSKVDLP---------------------DTIFHCGD------------QKYFPGLYG : 534
ApCPAP1-L  : ----QGTRRPVGNYPGQGLTSGFPGSRYPGQSGTIGFGPG------SHGIGSDDTGAYYNGDYSAIPGQPDIDYPIHSFIP---------------------HTSFSCN-------------SQTYPGYYA : 218
ApCPAP1-M  : ----------------------------------------------PCYYKKKPRVDFLYISFFYFSLLLLFQYLDFDNLP---------------------ETNFTCT-------------GKVIGGYYA : 236
ApCPAP1-O  : ----------------------------------------------GRGSQHLDHNGHGWDIRLAVPGDPGNDYPTLSSIP---------------------RTTFSCA--------------GREPGYYA :  64
ApCPAP1-B2 : FTKYEFSCSDFTVWDDDIQACNHPWAVRRSRCGKSGSNIDKKLGNQNETNVKETQSQIINKNDVTQTQLPTDHSNQLPQAPTTIT----------DSS-TKSSSSFYNNEDKYLTHINAQNSNQCTYSGFI : 318
BmCPAP1-A  : ----------------------------------------------QALIRKPLREHEKPQDLRNVPGTPGVDYPIYHSVP---------------------ETRFSCEH------------VPIHPGMYA :  91
BmCPAP1-B  : SSPVEQEYARPLPVANNYENEDVEVETYLDPVHPPSQHLDDYPL--HGSTSQQFPLPVTSDEEGGTPGSPGIDYPILTAIP---------------------QTSFNCK-------------TQRYKGFFA : 737
BmCPAP1-C  : -------------------------------------------------------------------------DTPQVKIP---------------------PTSFTCR--------------GRAAGYYA :  47
BmCPAP1-D  : ----------------------------------------------MGVAAVLSQQAIDYYHVLHLPHHPPL-HSVYDHAP---------------------PTRFSCA--------------GRQKGYYA :  59
BmCPAP1-F  : ----------------------------------------------VGFLAVFLYLIDKSIQQAIDDYVPGEDYPAFTEVP--------------------KGLSFTCN--------------DKIPGYYA :  64
BmCPAP1-G  : ----YQQRPQQQEQQEYVVPTRQQQQVYIEEPRAPVRAVQRKP---QEQRPLKYQQQQLQEVEEELEDEKEEPDRLSELLQ---------------------QSKFNCV--------------GKQTGYYA : 165
BmCPAP1-H  : ----------------------------------------------AETETTADGGEAPTNSTRKLSGIPQIDYLLDPNLPRELNGYNLSEYPFYEAVPPPETMDFKCD--------------GLHDGFYA : 129
BmCPAP1-I  : -----------------------------------------------MRTGGRRMRTSSQFLRDAHRENMSPLWILLTLLP-----------------VLGGAERFNCK-------------GRILGAYYA :  54
BmCPAP1-J  : ----------------------------------------------KRLIPADSHPDLDV---QGVIGKAGVDFPAYPNIP---------------------STEFNCK--------------NVPTGYYA : 122
BmCPAP1-K  : -------------TTSSTTTTTQPPVVFQPEPEILTAALPQKPQSTRQPTSGKVMNAPREYYPVGYEKNFDDHFQSKVDLP---------------------DTSFHCGD------------QKYFPGLYG : 466
BmCPAP1-L  : ----QESSGNYPGQDTRGPSSSFPGSRFPGRPGSPSFNPGSF----SPAFGTDGTGAYEGGDYSAIPGQPDIDYPILSDIP---------------------QTSFSCN-------------SQQYPGYYA : 140
BmCPAP1-M  : ----------------------------------------------SALQVWLWLLIFIFQNEFRISRSIQPGYLDFDNLP---------------------ETNFTCA-------------GKVIGGYYA :  83
BmCPAP1-N  : ----------------------------------------------DTNIMLKILRDMANQTKADTLNLPANATSIRENIT----------------------DTFSCE--------------NRTYGYYA :  77
BmCPAP1-O  : ----------------------------------------------GRGLQQHENNRHGWDIRIAVPGSPGEDYPTLSNIP---------------------RTPFSCA--------------GREPGYYA :  62
                                                                                                                        C
```

ChtDB2

```
ApCPAP1-A  : NVETGCQAYHVCHDGREG-----HQGASFLCTNGTLFDQAKFACDWW-YNVDCSQAIEHYKLNADPL---KNPYVPKRKPEPELVHEEPQQYHVPHGIFFRNV---------------------------- : 185
ApCPAP1-B  : DPETRCQVWHYCDLNGG--------QASFLCPNGTIFSQAGLTCDWW-FNVRCASTTQLYVLNESLY---KYILPHSPKFPEDFSGPL------------------------------------------- : 183
ApCPAP1-C  : EIKILHKVYHMCDGLGR--------QFSYTCPNTTLFQQRMLICDHW-YMVNCSMSERDYKANLLIG---QRDKPFVSDEEMGQRTPRPDILSVPLNNNYYDGLKE------------------------- :  95
ApCPAP1-D  : DVQSGCQAFHFCWRQRL--------VNTDLCTNGTLFNEQFQVCDHF-YNVRCGSPLEDL----------------------------------------------------------------------- :  94
ApCPAP1-F  : DTETSCQVWHWCVPSIGG-----NEKYSFLCAAGTAFNQRTRVCDWF-FKVDCQNSPGYYGINEDLY---KDESGNYISGKK------------------------------------------------- : 137
ApCPAP1-I  : DTKSGCKAFHVCVRVAGG----GVRDFRFYCPPGTLFHQEAQTCTDGGDDDPLACPADIYDGQFDLYKIGSGFDTKKLSSPGNREEETEYPLQQAETGDRRLSQNNIGHNSQASDLRAAHSS--------- : 190
ApCPAP1-J  : DLETDCQVFHICDTSRK---------ISFLCPNGTIFSQSHLICDWW-FKVDCASSPALYESSAEYY---SNEQKKSQKITHTLTKNHDLQQIGDSNIRSESRRSSI------------------------ : 210
ApCPAP1-K  : DESLGCMVFHVCALTDDG-----LVMKSFLCPESTLFDQTILKCNWW-FYVDCKNTRKLYDTNIPVS---KSYQLMKALTFFSSYKKE--MHDDGRPTNPEDVQGI------------------------- : 629
ApCPAP1-L  : DVETRCQVFHVCANNKT---------YDFLCPNGTVFSQEVFVCIWW-DQFDCNSAPSLYGLNANLY---DYSIIGSSGSFPSSGFNRPQGP--------------------------------------- : 297
ApCPAP1-M  : DLETSCQMFHVCTVGQQD----EPMDIKFLCLNGTVFDQETRVCERV-DEVDCTKSEKFYSLNLELY---GSTTPPIIQPEEPKPQTQPTEQTHPQTKPNQEQ---------------------------- : 331
ApCPAP1-O  : DTETNCQVFRVCTIGST------YGFQSFLCPNGTLFNQAVFVCDWW-MNVNCENTDQFVRNNNDKF---ENLRLGPQLMKDIKKMLTHPMRNPYDKIAMK------------------------------ : 155
ApCPAP1-B2 : GDPNNCKIFYRCVDNGKG----GYTRYEFSCGEGTFWDQNLEACNHAWAVKHCGSEQSLQETEITSP---SATKLSSSTIKPTQSYITSTKTTEKHKFEETDDNDKNVDYNDHNPDDLTTTLSHTLFTTTV : 442
BmCPAP1-A  : NVETGCQAYHVCHDGREG-----HQGASFLCTNGTLFDQTKFACDWW-YNVDCSQAIEHYKLNADPL---KNPYVPKQK--PEIHQEQPEEYNIPEEAYFRKY---------------------------- : 183
BmCPAP1-B  : DTETRCQVWHYCDLNGG--------QASFLCPNGTIFSQAGLTCDWW-FNVRCDSATQLYVLNESLY---KFILPHRPKFPEDYSGPL------------------------------------------- : 813
BmCPAP1-C  : DMETGCQVYHMCDGLGR--------RFSYSCPKTTLFQQRMLVCDHW-YMVNCSMAERDYDANLLIG---QRDKPFVSDEEMSQRTPRPDILSVPLTSKYYDGLKE------------------------- : 141
BmCPAP1-D  : DIDSGCQAYHFCWRQRL--------ISTDLCSKGTLFNEQFQVCDHF-YNVRCGSPYEDL----------------------------------------------------------------------- : 110
BmCPAP1-F  : DPETNCQVWHWCVPGIGG-----NQQYSFLCGIGTVFNQRTRVCDYF-FKVDCPNSPAYYGVNEDLY---KDEAGNYISGK-------------------------------------------------- : 136
BmCPAP1-G  : DEELNCEVFHYCQDSVK---------HSWICPDGFTFHQVHLICMPPTHDNICQKSSKYHFVNEYLY---RPVNQDEVDKKPNVSLKYSDRYYPAEVYRDDRYEQD------------------------- : 259
BmCPAP1-H  : SVPHKCQVYHHCLFGTR---------YDFLCANYTAFDQKTFICHFV-SEVDCKNSPNYFNRNEALY---KATTSAPPPPPPTTTTTTTAAPPPRAPRPARRRPHYRYDYYDDDYYYRDDYDYDDRRRPRP : 247
BmCPAP1-I  : DAKSGCKAFHVCVRVAGG----GIRDFRFFCPPGTLFHQEAQTCTDWGDDDPLACPADIYD----------GYDTRKLSSPANREEETEFGLQRAETGDRRLSQNNGASNGAGSDLRAAHSS--------- : 162
BmCPAP1-J  : DLETDCQVFHICDTSRK---------ISFLCPNGTIFSQSHLICDWW-FKVDCASAPALYESSAEYY---SNEQKKSQKTSQN-HKQQDLQLIGDSQIRAESRRSTV------------------------ : 215
BmCPAP1-K  : DESLGCMVFHVCALTDDG-----LVMKSFLCPESTLFDQTILKCNWW-FYVDCKNTRKLYDTNIPVS---KSYQLMKALTFFSSYKKDNNMQDDGRSPNPEDVDGI------------------------- : 563
BmCPAP1-L  : DVETRCQVFHVCANNKT---------YDFLCPNGTVFSQEVFVCVWW-NQFDCSSAPSLFSLNANLY---DYSITGSQGGFPSSGLNNYPGGQRGQTFPGRGPLRP------------------------- : 233
BmCPAP1-M  : DLETSCQMFHVCTVGQQD----EPMDIKFLCLNGTVFDQETRVCERV-DEVDCTKSEKFYSLNLELY---GSTAPPIIQPEQNKSETTNNKRENE------------------------------------ : 170
BmCPAP1-N  : DIDNDCQVFHVCLPTQTPSGRAVTYRYSFICPSETVFNQEVLTCTRPRDAIECEDSVLYYDLNEEIG---KVPNKTEEQPPVNAQQPAKSSSQQKETETLPATNQRRTPNKRKQSLIIESLM--------- : 196
BmCPAP1-O  : DFETNCQVFRVCTVGST------YGFQSFLCPNGTLFNQAVFVCDWW-MNVNCRESEKLLNSKNEQF---QNLRLGPELMKDIKKMLTYPMRNPYNKASMR------------------------------ : 153
                  C     C                  C            C        C
```
