# Supplementary material for: Genome-Wide Identification and Transcriptome-Based Expression Profile of Cuticular Protein Genes in Antheraea pernyi
Source: Int J Mol Sci. 2023 Apr 10;24(8):6991. doi: 10.3390/ijms24086991 (PMC10138643; doi:10.3390/ijms24086991)
Supplement: Supplementary file 1 [file ijms-24-06991-s001.zip › Figure S4.pdf]

CPAP3:

```
ApCPAP3-A1 : --------------------------------------------------------------------------------------ALASAQ------------------------YK--CPNKDGQ--YED :  29
ApCPAP3-A2 : --------------------------------------------------------------------------------------GL--GQ-----------------------DFN--CPEKSGF--YPD :  30
ApCPAP3-B  : --------------------------------------------------------------------------------------VLVSSAVPRGKKKPANPPARVIEESEKDAEITSSCPE-DGF--YAD :  59
ApCPAP3-C  : --------------------------------------------------------------------------------------ILTVAQE----------------------SFK--CPDDFGF--YPH :  25
ApCPAP3-D2 : --------------------------------------------------------------------------------------LTSCTQV----------------------EDP--CKTKARV--VAD :  36
ApCPAP3-E  : --------------------------------------------------------------------------------------ALAYAQSEYDDSEAESSQA-NSQYQQTSTNLVGACKELNER--YAV :  55
BmCPAP3-A1 : --------------------------------------------------------------------------------------AIASAQ------------------------FK--CPAKDGQ--YED :  29
BmCPAP3-A2 : --------------------------------------------------------------------------------------GLVSGQ-----------------------EFK--CPDKSGF--YPD :  31
BmCPAP3-B  : --------------------------------------------------------------------------------------VVTSAQSPKARKKPANPPARAREESEADPEITNLCPE-DGY--FAD :  58
BmCPAP3-C  : --------------------------------------------------------------------------------------GVAVAQE----------------------SFK--CPDDFGF--YPH :  33
BmCPAP3-D1 : --------------------------------------------------------------------------------------AFANAGILLEH------------------APP--CPEEYGVQAYAH :  38
BmCPAP3-D2 : --------------------------------------------------------------------------------------LTSCAQI----------------------EDP--CKTKARI--VAD :  37
BmCPAP3-E  : --------------------------------------------------------------------------------------AFVYTQTQYQPFGQTNRFQ-NQYQEQAPVRPTGQCRELNER--YPV :  55
BmCPAP3-E2 : --------------------------------------------------------------------------------------AASHVQS--------------TPAAATAQNVI--CKARNGY--YKT :  39
BmCPAP3-E4 : ITPRPVPVLISQPTEFLTTTTPLSELQPVSAQPAVPPISFVTQETETRLEPHPIQIPKPNPQPAPVLLPVSPPTPYPNIWSQPPQPTLFPAQKPIDVRQPPPPMT-YAASYSKTTDFV--CREAYGY--YPV : 523
                                                                                                                                    C
```

ChtDB2 ChtDB2

```
ApCPAP3-A1 : ERQCDKFYECVDGVSTTKLCPDGLVFD--PTIRKINKCDQPFNVDCGD--RTELQPPKPNSLCPRRNGFFAHPD--SSVCN-IFFNCIEGDAIEVKCTAGLHFDEYSGTCVWPDSAGRQGCV--EQEKKTKD : 152
ApCPAP3-A2 : PYQCDLYYKCSKGQSEAKLCPDGLVFL--DENPSKEHCDIPSNVDCGD--RKELQEPKPSTGCPRQNGYYKHPD--PQACD-KFYYCSDGVPNELPCPPGLYFDEETSNCDWKESVNRQ-CDKITKDVL-DD : 153
ApCPAP3-B  : AEQCDKYYACQEGQITEKLCPDGMVFN--DYNVQDEKCDLPFNIDCSQ--RPNLQTPIPSLHCPRKNGYFAHDD--PKICG-IFYYCVDGKFNMITCPSGLVYNDKTGICTWADEAKKEGCG--ADEVF--- : 179
ApCPAP3-C  : HISCDKYWKCDNGVAELKTCGNGLAFDATDSKYLTENCDYLHNVECGE--RTQLEPPISTPHCSRLYGIF--PD--EAKCD-VFWNCWNGEASRYQCSPGLAYDRESRVCMWADQVPE--CR--NEEVA--N : 144
ApCPAP3-D2 : DKHCDKYWECDNGQSVQYDCPNGLVFAG-KHRGVTEGCDYPWRSNYCEYPKVQTNPPIGVEHCDWLYGIFGH----ETSCT-RYWTCWNGTATEQLCIGGLLYNENAHSCDWPENVDG--CQ--KHPL---- : 154
ApCPAP3-E  : PGSCDKYIECINGTAEEKLCPDGLRFNR-NVNFNVYPCQYPNEVTCLE--RSALQPAQPTELCPHQFGYFRIGD--AKNCS-GFRNCVNGVGYDFVCPEGLAFSPDNYRCDWPDQVAE--CD--AEAFL--- : 174
BmCPAP3-A1 : DRQCDKFFECVDGVATTKLCPDGLVFD--PTIRKINKCDQPFNVDCGD--RTELQPPKPNSQCPRRNGFFAHPD--PSVCN-IFYNCIEGEATEVKCTAGLHFDEYSGTCVWPDSAGRQGCN--EQQKKTKD : 152
BmCPAP3-A2 : PYQCDLYYKCSRGDAEEKLCPDGLVFS--DENPNKEHCDIPSNVDCGD--RKELQEPKPSKGCPRQNGYFKHPD--PQACD-KFHYCADGIPNELPCPPGLYFDEETSNCDWKEVVNRQ-CDQITKDVL-DD : 154
BmCPAP3-B  : AEQCDKYYECRGGDIIEKLCPDGMVFN--DYSPQEEKCDLPFNLDCSQ--RPKLQTPQPSLHCIRQNGYFSHED--PKECG-KFYFCVDGKFNMITCPDGLVYNDKTGICTWPDEAKKKGCG--AAEVF--- : 178
BmCPAP3-C  : HISCDKYWKCDNGVAELKTCGNGLAFDATDSKYLTENCDYLHNVECGE--RTQLEPPISTPHCSRLYGIF--PD--ENKCD-VFWNCWNGEASRYQCSPGLAYDRESRVCMWADQVPE--CK--NEEVA--N : 152
BmCPAP3-D1 : PEQCDQFFLCTNGTLTVETCENGLLFD--GKGAVHNHCNYNWAVDCGH--RTANLEPLSTPGCEYQFGIY--PD--SHECSTSYIKCAYGVPEQFPCTPGLVYDERSHSCNWPDLLQPF-CN--PEAVV--- : 156
BmCPAP3-D2 : DKYCDKYWECDNGQSVQYDCPNGLVFAG-KHRGVTEGCDYPWRSNYCEYPKAQINPPIGTEHCDWLYGIFGH----ETSCT-RYWTCWNGTATEQLCIGGLLYNENAHSCDWPENVDG--CQ--KHPL---- : 155
BmCPAP3-E  : SGSCDRYIECINGTAEEKLCPDGLRYNP-NVNFDVYPCQYPNEVPCLE--RSSLQPPQPTELCPHQFGYFKLGD--ARNCS-GFRNCVNGVGYDFVCPDGLAFNSETYRCEWPDEVAD--CD--AEAFL--- : 174
BmCPAP3-E2 : DANCDTYIECRDYQATNMICPDGLHFNP-SVEWPAYPCGYPVEVTCVG--RGSIQPAQTTPDCPHQYGIFKHPNASPTNCG-QYRTCVGGRAFDMVCPPGLAFNPDFSRCDWADLVPS--CD--AEKFL--- : 160
BmCPAP3-E4 : TNECDTYIECKQGIAVKQSCPDGLHFKS-STQWPDYPCAYPSDVQCTS--GSIKQYANPTAECPHEYGSYPLK---ESNCS-NYIMCHEGKPTIMHCPYGLAFNFDKNSCDWPENVPA--CN--VDAFK--- : 641
                C     C          C                 C       C                C                C      C         C            C      C
```

ChtDB2

```
ApCPAP3-A1 : GFECPKE-QQVDAQGLPVAHPKFPHPNDCQRFYVCLNGIEPRDLGCQVGEVYN----EESQKCDAPENVRGCEDWYK-----------DSEEAAPGPKARS-------------------------------- : 237
ApCPAP3-A2 : GFSCPDG-EVMGPNGRALPHPTFPHPEDCQKFYICRNGVQPQKGSCPSGKVYN----EDNFMCDEPEKVTGCENYYEGQ---PLE-----------------------------KNKLPKKA---------- : 238
ApCPAP3-B  : QFQCPSV-----NESFALTHPRYADPDDCQYFYVCINGNTPRRSGCKLGQVFD----DVNKRCEWARKVPECADWYKGQ---LTDAELDALENPPTLKPKPAGT-----QPSRRKPQRPHKGKAVEAPPADE : 294
ApCPAP3-C  : GFACASP-GEVSNIG---SFSRHAHPEDCRKYYICLEG-VAREYGCPIGTVFKIGDADGTGNCEDPEDVPGCEDYYG-------DLDLKTIRKSELLAGLQSNGQTRTTQPKQLKPRPAKEN---------- : 254
ApCPAP3-D2 : ---CNED-----------PNGNVPLGKSCNRYWQCQGG-YPRLQRCPAMLVFD----RRSLRCVVPPTE-ECDIPTTTAPPPEEEQQQDTRQGQQSPQKRPSND-----FQETQS-RQRPRN---------- : 250
ApCPAP3-E  : GFRCPEI-PTSKELGAPAGYRFYRSDTNCQKYFICVDG-NPRLLYCGGDSAFD----ELTSTCVSADEVSECPEELRTH---AARTKEEEQQR--LAKELEFSE-----IKAKRK--LNKEENV-------- : 280
BmCPAP3-A1 : GFECPKE-QLVDAQGQIVAHPKFPHPNDCQRFYVCLNGVEPRDLGCQVGEVYN----EESQKCDAPENVRGCEDWYK-----------DSEEAAPGPKSRS-------------------------------- : 237
BmCPAP3-A2 : GFTCPDG-EVMGPNGRSLPHPTFPHPEDCQKFYICRNGVQPQKGSCPSGKVYN----EDTFMCDDPEKVVGCENYYDGQ---PLD-----------------------------KNKLPKKA---------- : 239
BmCPAP3-B  : QFECPAV-----NETFGLTHPRYADPDDCQFFYVCINGNTPRRSGCKLGQAFD----DVSKKCEWARKVPECAEWYKGQ---LTDAELDALETPPTPKPKPAGS------PSRRKPQRPSKGKTVQ---IED : 289
BmCPAP3-C  : GFGCPAP-GEVSNAG---SFSRHAHPEDCRKYYICLEG-VAREYGCPIGTVFKIGDADGTGNCEDPEDVPGCEDYYG-------ELDLKAIRKSELLAGLQADGQPRPNQPKQLKPRPPKEN---------- : 262
BmCPAP3-D1 : GFKCPSKVPANTPSAKFWPFPRFPVPGDCHRLITCVEG-QPRLITCEEGKVFD----DQNLTCEDPEIVPHC---------------------------------------------GRA---------- : 226
BmCPAP3-D2 : ---CNED-----------PNGNVPLGKSCNRYWQCQGG-YPRLQRCPAMLVFD----RRSLRCVVPPTE-DCDIPTTTAPPPEEE-QQDARQGQH--QKRPSND-----FQEQQQGRTRPRN---------- : 249
BmCPAP3-E  : GFRCPEV-PISRELGPPAGYRYYRSDNNCQKYFLCIDG-HPRVLYCGGESAFD----DLTSTCVSADEVGACPAELRAA---AVRAKDDAKQL--LAKEVEFNA-----YQLTRKPSLSYRN---------- : 280
BmCPAP3-E2 : GFTCPPA-PLDAIGNPLNNVINYKYEGNCYYFFSCEQN-RARLLSCDIGLAFD----PTTGRCVDADRV-QC----------------NATQN-------------------------TNDKNHI-------- : 237
BmCPAP3-E4 : NFTCPTP-PINEVGDLSDTMHKYRYGNSCSFYIVCHRG-YPRLLSCDPGLSFD----QESESCIDSALVPNC----------------NMGQK---------------------------------------- : 712
                C                           C    C         C              C       C
```
