# Supplementary material for: Genome-Wide Identification and Transcriptome-Based Expression Profile of Cuticular Protein Genes in Antheraea pernyi
Source: Int J Mol Sci. 2023 Apr 10;24(8):6991. doi: 10.3390/ijms24086991 (PMC10138643; doi:10.3390/ijms24086991)
Supplement: Supplementary file 1 [file ijms-24-06991-s001.zip › Figure S5.pdf]

```
ApCPF      : M---LLLFCCAMVAVHGGALISPVYGAPYSYGSWNPYSSYPSTPALASQHSNTYRSPFNLGQVSTYSKSVDTPFSSVRKADIRVSNPGVAVSPAYSGFAAPYVSHVGLAAPLTAPVAKVAT--GLLGVAYSAAP : 129
BmorCPF    : MILKIVLLCGAVAAVHGGGLIAPAYSAPYSYGAWNPYSSYPAQPALASQHSNT--SPFNLGQISTYSKSVDTPFSSVRKADIRVSNPGVAVAPAYSSFAAPYVSHVGVA----APVAKVATTGGLLGVAYSAAP : 128

ApCPF      : AVSHMTYTNGLGLAYACVARTNGLIQMYSIFNYFTDYNESLPFSAVVIAHSDKFEDVYDYFRAVLQRNEKSERALQLTKDAVELNPANYTVWQYRRDLLQALDTNLRLELDYVESVIKSQPKNYQVWHHRRVLV : 263
BmorCPF    : TVSHMTYTNGLGFAYG---------------------------------------------------------------------------W------------------------------------------ : 145
```

.
