# Supplementary material for: Genome-Wide Identification and Transcriptome-Based Expression Profile of Cuticular Protein Genes in Antheraea pernyi
Source: Int J Mol Sci. 2023 Apr 10;24(8):6991. doi: 10.3390/ijms24086991 (PMC10138643; doi:10.3390/ijms24086991)
Supplement: Supplementary file 1 [file ijms-24-06991-s001.zip › Supplementary Captions.pdf]

Figure S1: The exterior observation of larval epidermis and prothoracic gland of *Antheraea pernyi* strain Qing 6 and *Bombyx mori* strain P50.

Figures S2: Chromosomal localization of cuticular protein genes in *Bombyx mori*.

Figure S3: Multiple sequence alignment of *Antheraea pernyi* and *Bombyx mori* CPAP1.

Figure S4: Multiple sequence alignment of *Antheraea pernyi* and *Bombyx mori* CPAP3.

Figure S5: Multiple sequence alignment of *Antheraea pernyi* and *Bombyx mori* CPT.

Figure S6: The gene expression level of CP genes in the larval epidermis of *Antheraea pernyi* (A) and *Bombyx mori* (B).

Figure S7: The gene expression level of CP genes in the larval prothoracic gland of *Antheraea pernyi* (A) and *Bombyx mori* (B).

Table S1: Statistical analysis of the transcriptomic data for the epidermis, haemolymph and midgut in *Antheraea pernyi*.

Table S2: Information on the CPR, CPAP, CPF, CPFL, CPT, CPCFC, CPLCP, CPLCA, CPG, CPH, and 18 aa families in *Bombyx mori*.

Table S3: Information on the CPR, CPAP, CPF, CPFL, CPT, CPCFC, CPLCP, CPG and CPH families identified from *Antheraea pernyi*.

Table S4: Orthologous groups of CPR RR-2 genes between *Antheraea pernyi* and *Bombyx mori*.

Table S5: Information on sequence identity and chromosomal localization of orthologous CPs between *Antheraea pernyi* and *Bombyx mori*.

Table S6: FPKM values of cuticular protein genes in six tissues/organs of *Antheraea pernyi*.

Table S7: FPKM values of cuticular protein genes in three tissues/organs of *Bombyx mori*.
