# Supplementary figures and images for: Genome-Wide Identification and Transcriptome-Based Expression Profile of Cuticular Protein Genes in Antheraea pernyi
Source: Int J Mol Sci. 2023 Apr 10;24(8):6991. doi: 10.3390/ijms24086991 (PMC10138643; doi:10.3390/ijms24086991)

Epidermis

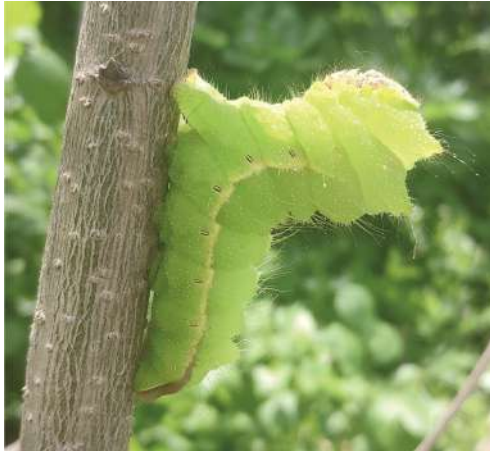

*A. pernyi*

Prothoracic gland

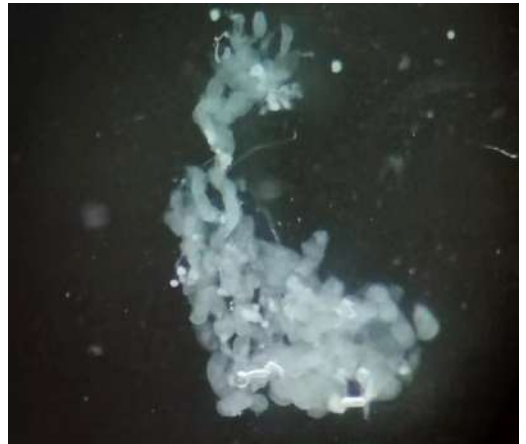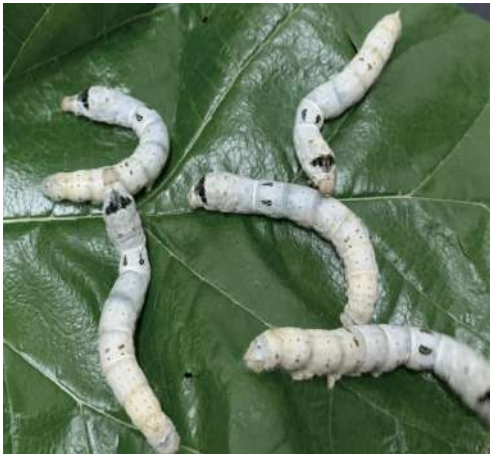

*B. mori*

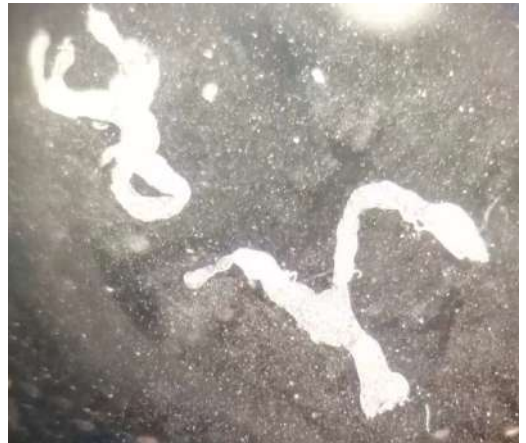

Supplement: Supplementary file 1 [file ijms-24-06991-s001.zip › Figure S1.pdf]

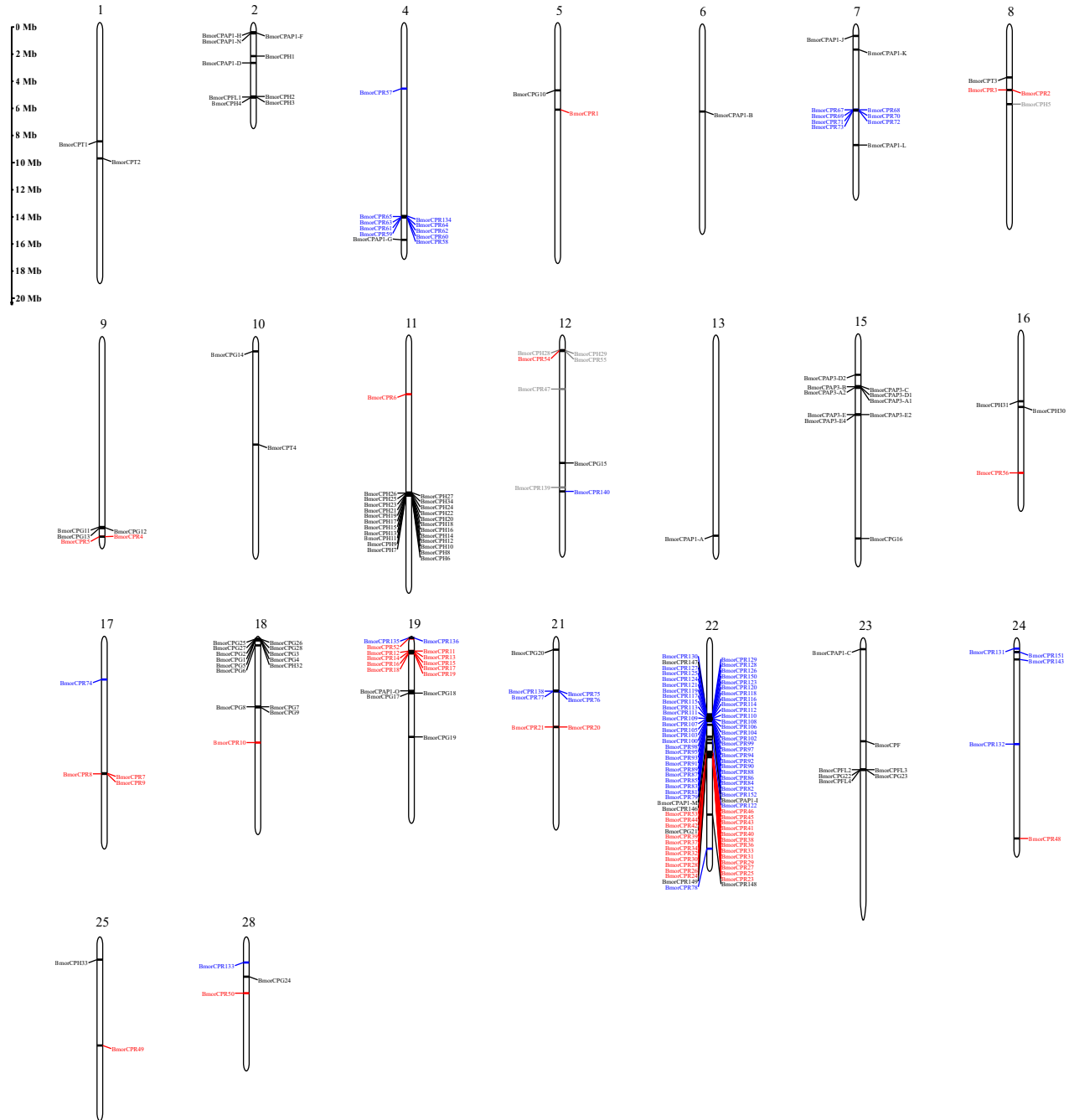

Supplement: Supplementary file 1 [file ijms-24-06991-s001.zip › Figure S2.pdf]

(A)

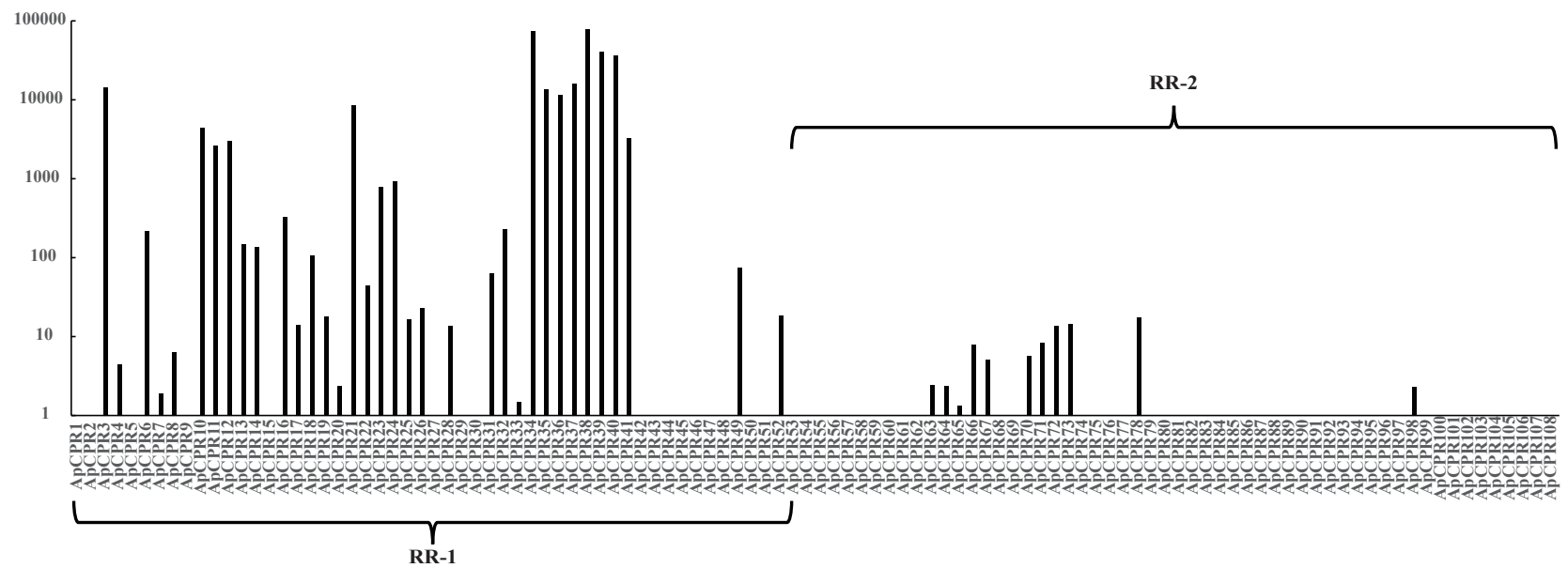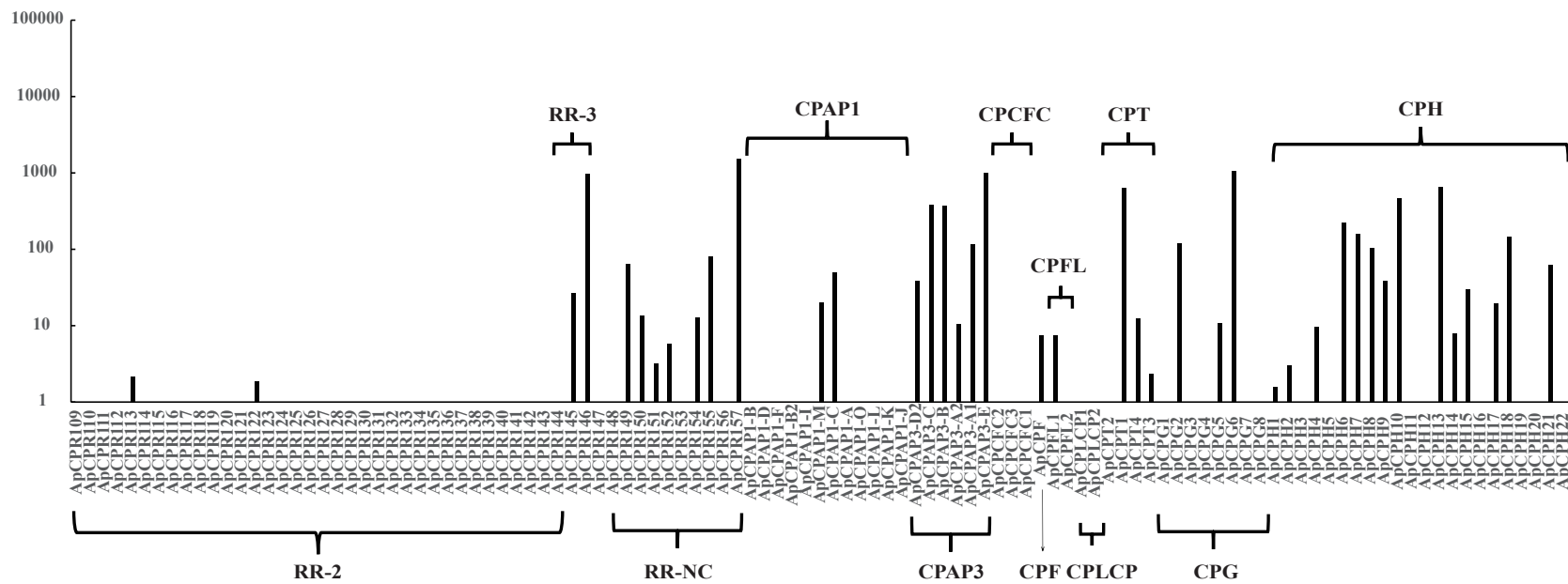

Supplement: Supplementary file 1 [file ijms-24-06991-s001.zip › Figure S6(A).pdf]

(B)

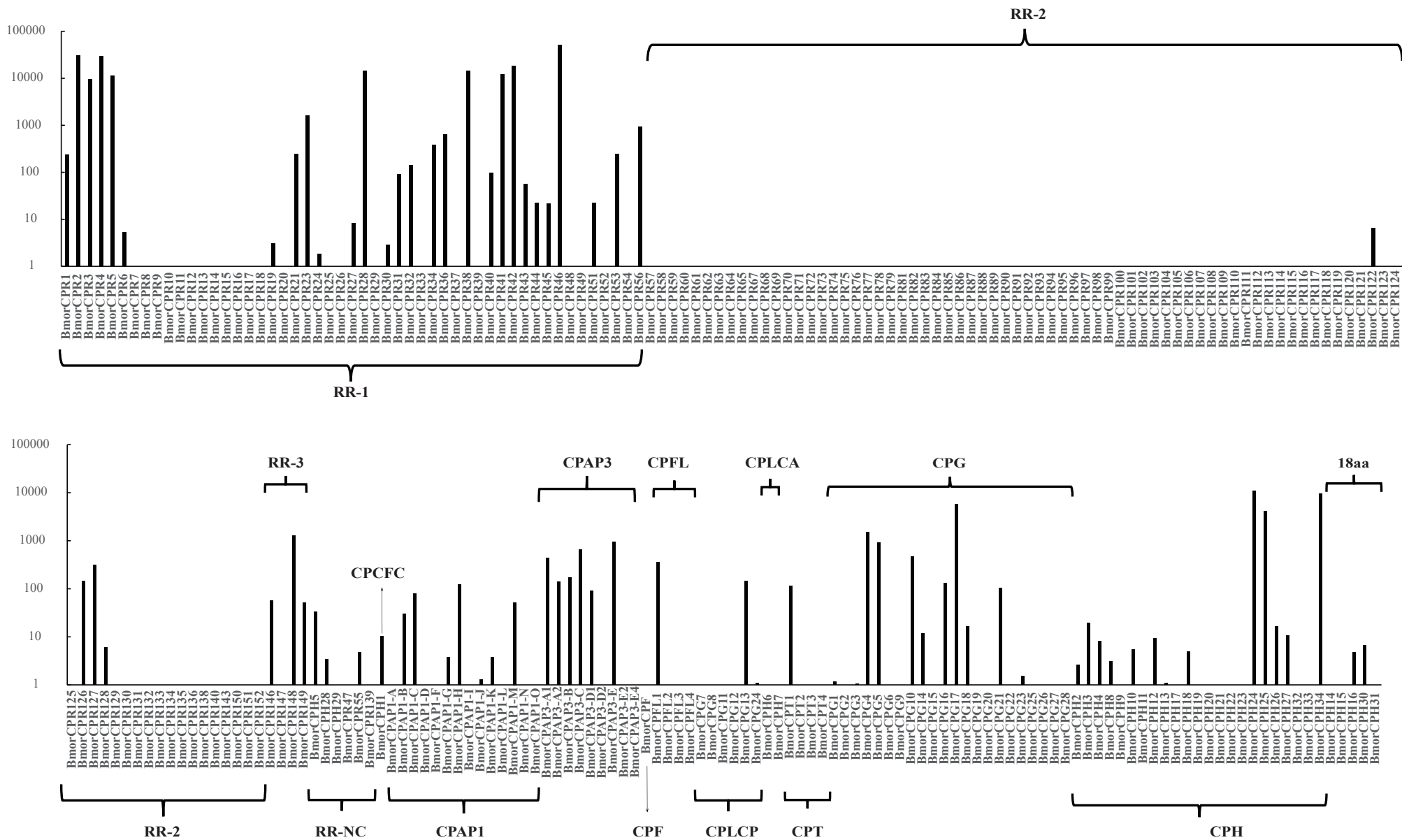

Supplement: Supplementary file 1 [file ijms-24-06991-s001.zip › Figure S6(B).pdf]

(A)

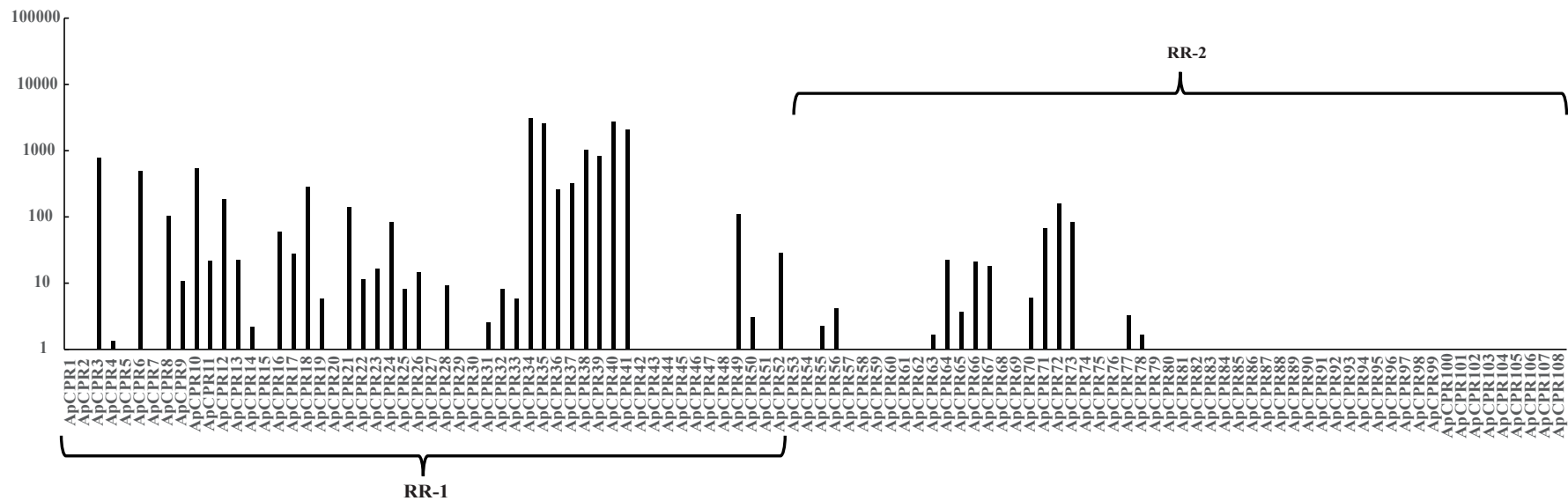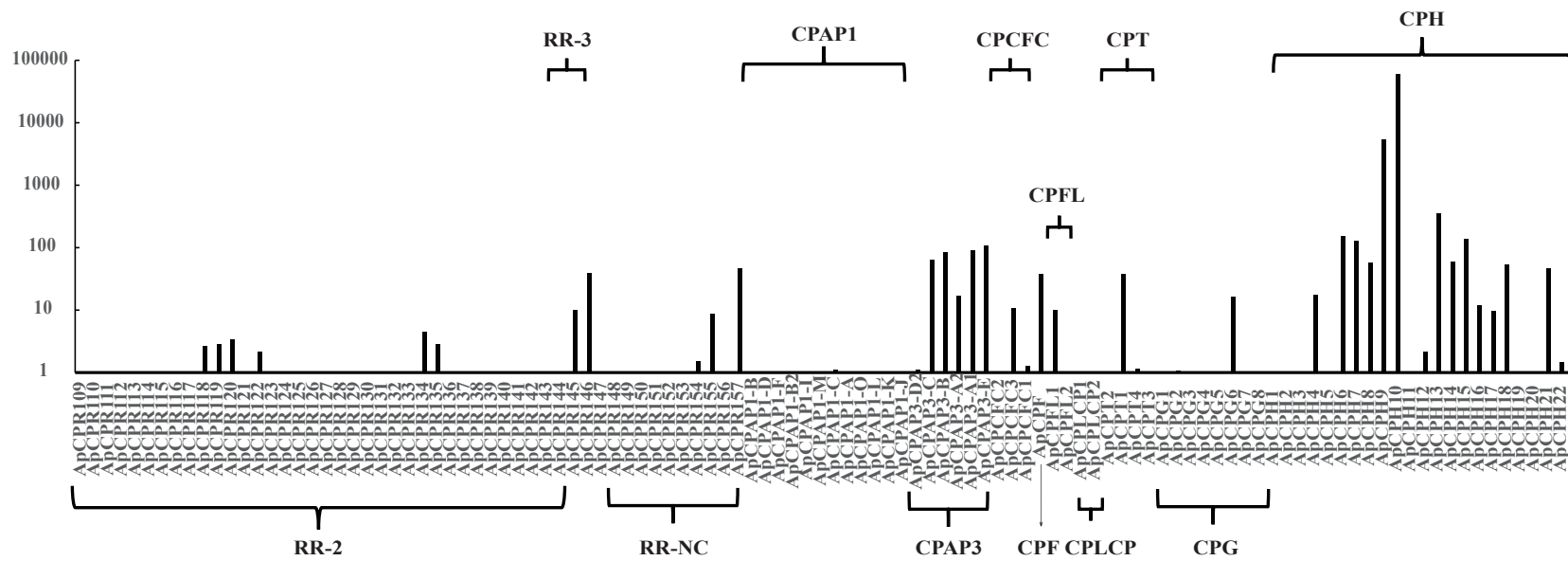

Supplement: Supplementary file 1 [file ijms-24-06991-s001.zip › Figure S7(A).pdf]

(B)

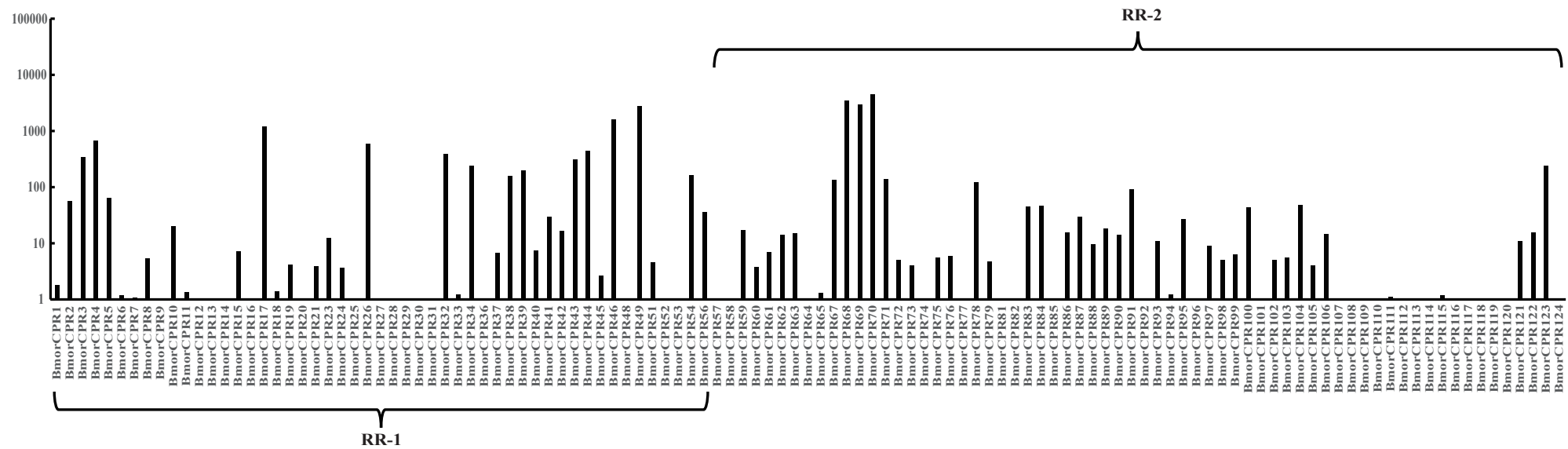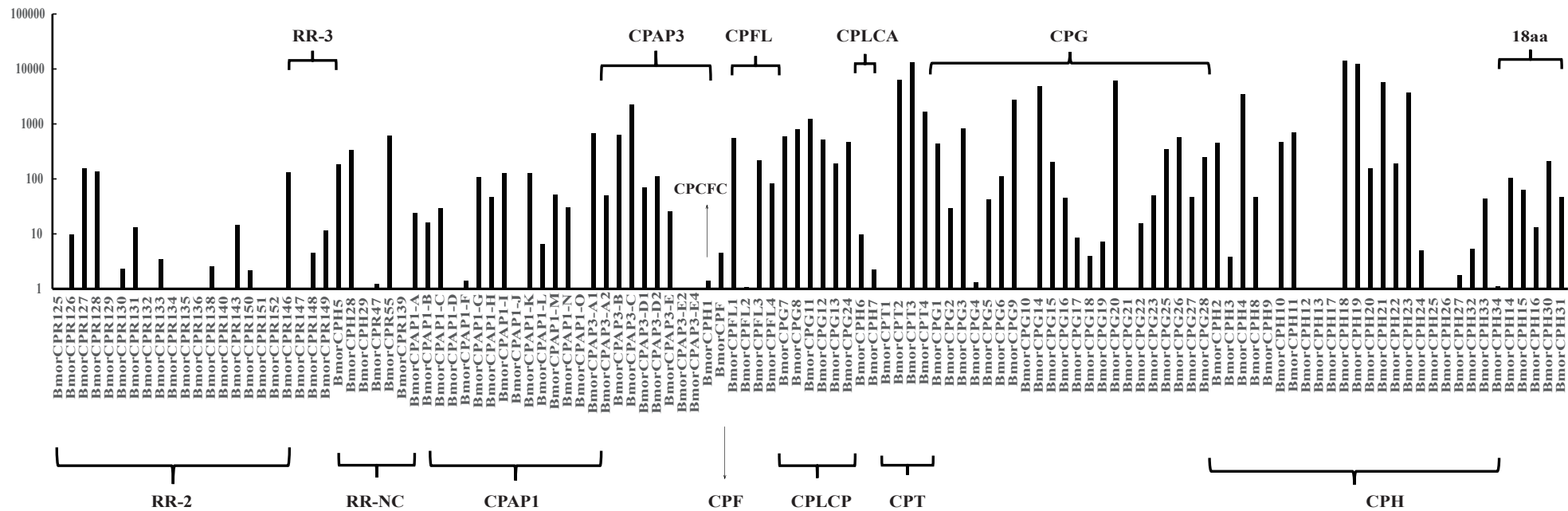

Supplement: Supplementary file 1 [file ijms-24-06991-s001.zip › Figure S7(B).pdf]
